# Effects of short-term fructooligosaccharide intake on equol production in Japanese postmenopausal women consuming soy isoflavone supplements: a pilot study

**DOI:** 10.1186/1475-2891-12-127

**Published:** 2013-09-13

**Authors:** Yuko Tousen, Mariko Uehara, Fumiko Abe, Yoshifumi Kimira, Yoshiko Ishimi

**Affiliations:** 1 Department of Food Function and Labeling, National Institute of Health and Nutrition, 1-23-1 Toyama, Shinjuku-ku, Tokyo 162-8636, Japan; 2 Department of Nutritional Science, Faculty of Applied Bio-Science, Tokyo University of Agriculture, 1-1-1 Sakuragaoka, Setagaya-ku, Tokyo 156-8502, Japan; 3 Department of Clinical Dietetics & Human Nutrition, Faculty of Pharmaceutical Sciences, Josai University, 1-1 Keyakidai, Sakado, Saitama 350-0295, Japan

**Keywords:** Isoflavones, Equol, Fructooligosaccharides, Intestinal microbiota

## Abstract

**Background:**

Recent studies suggest that some of the clinical effectiveness of soy or daidzein, which is a type of isoflavone, may be attributed to a person’s ability to produce equol from daidzein. Equol, which is a metabolite of one of the major soybean isoflavones called daidzein, is produced in the gastrointestinal tract by certain intestinal microbiota where present. Habitual dietary patterns may alter the intestinal bacterial profile, and influence the metabolism of isoflavones and the production of equol. Fructooligosaccharides (FOS) have a prebiotic activity as well as being a dietary fibre. The purpose of the present study was to determine whether FOS supplementation increases equol production in equol producers and stimulates equol production in equol non-producers in Japanese postmenopausal women.

**Methods:**

A soy challenge was used to assess equol-producer status prior to the start of the study in healthy postmenopausal Japanese women. The study involved 4 separate groups in randomised crossover design. First, subjects were classified as equol producers (n = 25) or non-producers (n = 18), and then they were randomly assigned to the FOS or control group. All subjects received a daily dose of 37 mg isoflavone conjugates in the capsule (21 mg aglycone form) and either FOS (5g/day) or sucrose as control, in a randomised crossover study design. Equol -production was assessed by testing the serum and urine before and after the 2-week supplementation period.

**Results:**

The analyses were conducted on 34 subjects completed the study, 21 (61.8%) were classified as equol producers, and 13 (38.2%) as non-producers. Significant differences were observed in the interaction effect of time × equol state after 1 week of intervention (p = 0.006). However there were no effects after 2 weeks of intervention (p = 0.516). Finally, in both equol producers and non-producers, FOS supplementation did not affect the serum equol concentration or the urinary equol to daidzein concentration ratios.

**Conclusions:**

We have reported that FOS intervention (5 g/day for 2 weeks) does not significantly modulate the capacity of intestinal microbiota to produce equol in postmenopausal Japanese women, in either equol producers or non-producers in this pilot study. Further larger investigations that explore the roles of specific intestinal microbiota in equol production will enable the establishment of dietary conditions that are required to enhance equol production.

## Background

Soybean isoflavones are structurally similar to oestrogen; they exhibit a weak affinity for oestrogen receptors, ERα and ERβ, eliciting mild oestrogenic-like activity in various tissues [1]. On the other hand, the isoflavones genistein and daidzein have been shown previously to possess anti-oestrogenic activity in human breast cancer cells in vitro [2]. Compared with ERα, ERβ exhibits a greater binding affinity for isoflavone, whereas oestrogen binds to ER with equal affinity [3]. The relatively selective binding of isoflavones to ERβ indicates that isoflavones may confer distinct clinical effects compared with oestrogen [4]. Moreover Hwang et al. reported that isoflavones may exert their effects as oestrogen antagonists in a high oestrogen environment, or they may act as oestrogen agonists in a low oestrogen environment [5].

Isoflavones have received considerable attention because of their potential to prevent postmenopausal conditions such as cardiovascular disease [6], osteoporosis [7], and hormone-dependent cancers [8]. Daidzein, a major soybean isoflavone, is metabolized to equol in the gastrointestinal tract by intestinal microbiota. Recent studies suggest that some of the clinical effectiveness of soy or daidzein, which is a type of isoflavone, can be attributed to a person’s ability to produce equol from daidzein via their intestinal bacteria, because its biological activities differ from those of its precursor [9]. However, not all healthy humans produce equol. The ability to produce equol depends on the presence of certain intestinal microbiota. Many studies have provided that only 25-30% of the adult population of Western countries produce equol when fed soy foods containing isoflavones [10-12]. This is significantly lower than the reported 50-60% of equol producers in adults from Asian countries [13-15]. The reasons for these differences are unclear, but dietary changes can alter the microbiotal profile of the intestine. Therefore, habitual dietary patterns may influence the metabolism of isoflavone and the production of equol [16]. Several studies comparing the habitual diets of equol producers and non-producers who consume Western diets have reported that equol producers tend to have a higher intake of carbohydrate and dietary fibre, a higher percentage of energy as carbohydrate and lower percentage of energy as fat, and intake of soy, plant protein [10,12,17]; however, this has not been a consistent observation.

On the other hand, soy products are traditionally used in many Asian countries. The intake of traditional soy-based foods is high in Japan, and the mean total intake of isoflavones is estimated between 19.4 and 33.6 mg/d according to the National Nutritional Survey in Japan [18]. In Western populations, the consumption of isoflavones from traditional soy foods is substantially lower (between 0.5 and 3 mg) than that of Japan [19-21]. In Western countries, a portion of total isoflavone consumption is derived from soy protein and soy flour added to a variety of foods.

While it is not clearly understood how bacterial species produce equol in the intestine, certain *Lactobacillus* and *Bifidobacteria* species have been suggested to play a role in the metabolism of daidzein to equol [22,23]. Fructooligosaccharides (FOS) have prebiotic activity in addition to being a form of dietary fibre [24,25]. FOS are poorly digested in the human small intestine and are fermented in the colon by *Lactobacillus* and *Bifidobacteria* species; moreover, FOS stimulates their growth.

To date, a few studies have reported the effects of dietary fibre and/or prebiotics on isoflavone bioavailability in Western population [26-28], but no intervention studies have examined the effect of FOS on equol production in Japanese. The purpose of the present study was to determine whether FOS supplementation increases equol production in equol producers and stimulates equol production in equol non-producers in Japanese postmenopausal women.

## Methods

### Ethics statement

The protocol was approved by the Institutional Review Board of the National Institute of Health and Nutrition of Japan, and the study was performed in accordance with the guidelines of the Declaration of Helsinki. All women provided their written informed consent to participate in the study.

### Subjects and design

In this study, 43 healthy postmenopausal women who fulfilled the required criteria were recruited and screened. The exclusion criteria for this study were history of chronic renal or hepatic disease, history of hormone replacement therapy or breast cancer, serum oestrogen hormone (E2) concentration > 50 pg/mL, soy allergies, and any other medication known to affect bone. A soy challenge was used to assess equol-producer status prior to the start of this study. The day before sample collection, subjects ingested soy food containing 22 mg soy isoflavones. On sample collection day, morning urine samples were collected from the subjects, and they were stratified into equol producers or non-producers based on their urinary equol to daidzein concentration ratio [Equol producer: Log (urinary equol/daidzein) > −1.70].

**Figure 1:**
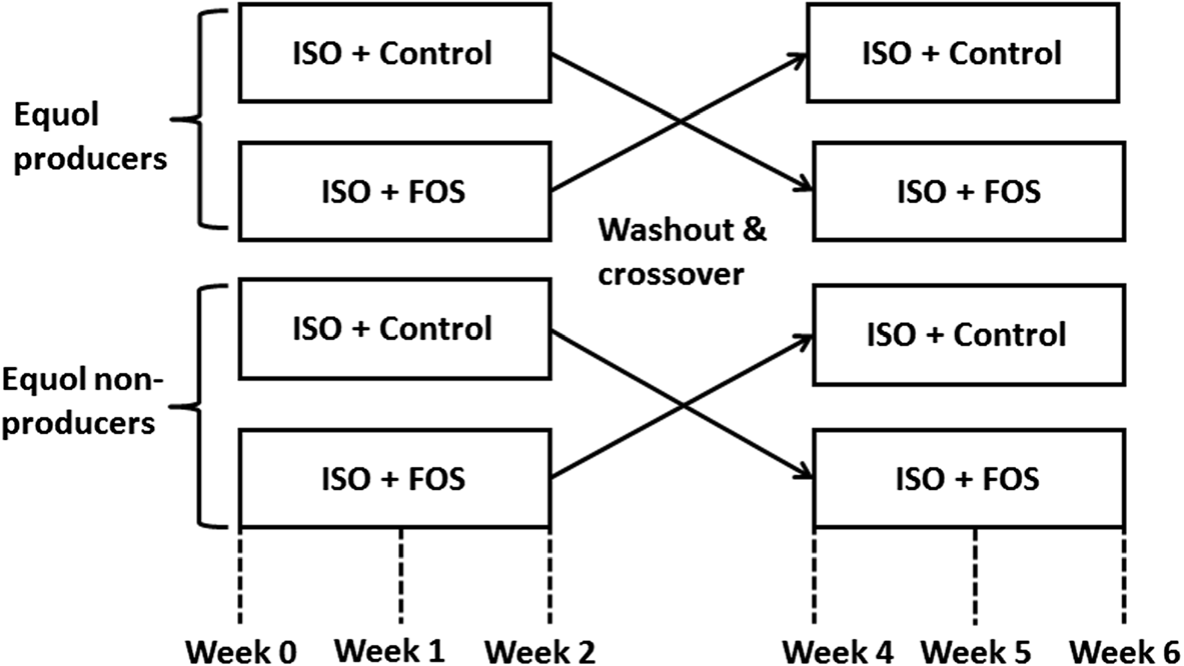
**Study design.** Subjects were classified as equol producers or non-producers and were further assigned to 2 groups as follows: ISO + control, intervention with soy isoflavone supplement and sucrose; ISO + FOS, intervention with soy isoflavone supplement and fructooligosaccharides. ISO, isoflavones; FOS, fructooligosaccharides.

The study comprises 4 separate groups in a randomized crossover design (Figure 1). Subjects were identified by a single randomisation number using a computer-generated random permutation procedure in SPSS software version 11.0J. The study design involved two 2-week dietary periods separated by a 2-week washout. During each 2-week intervention period, fermented foods and additional soy isoflavone supplements that could potentially affect intestinal bacteria were excluded from the diet. Subjects were instructed to otherwise maintain their normal diets for the duration of the study. The washout period required the same dietary exclusions as the intervention periods. Studies have shown that antibiotic-associated changes in faecal flora are normally reversed in 10–14 days after the stopping of treatment [29,30]. Therefore, a 2-week washout period was set in our study, although antibiotics were not used by any of the participants during the study. The subject randomisation codes were allocated sequentially in the order in which the subjects were enrolled. After completion of all the analyses, the randomisation code was disclosed to the investigators and subjects.

All subjects received a daily dose of 37 mg isoflavone conjugates in the capsule (Fujiflavone P40; Fujicco Co., Ltd., Kobe, Japan; 21 mg aglycone form) and 5 g of either FOS (Meioligo; Meiji Seika Kaisha Ltd., Tokyo, Japan) or sucrose (white sugar; Mitsui Sugar Co., Ltd., Tokyo, Japan) as control. Each subject consumed the isoflavone capsule and FOS or sucrose at breakfast. Digestive products may stimulate the growth of certain microbiota, and FOS may selectively stimulate the growth of certain microbiota. As sucrose is digested readily, we used that as the control in this study. Dietary assessments of dietary soy isoflavones and nutrient intake based on 3-day dietary records were obtained at baseline. Fasting blood samples were collected at baseline and after 2 weeks of intervention. Spot urinary samples were collected at baseline and after 1 and 2 weeks of intervention.

### Isoflavones supplement

The crude isoflavone conjugate (40% isoflavones) in the capsule were daidzin (20.4%), malonyldaidzin (0.1%), acetyldaidzin (1.1%), daidzein (0.3%), genistin (4.6%), acetylgenistin (0.3%), genistein (0.1%), and glycitin plus glycitein (13%). As aglycones, 33 mg daidzein, 8.5 mg genistein, and 15 mg glycitein were included in the 100 mg of conjugates. Thus, 37 mg of conjugated isoflavones equivalent to 21.1 mg aglycone, which consisted of 12.3 mg daidzein, 3.2 mg genistein, and 5.6 mg glycitein, existed in each capsule.

### Measurement of serum and urinary daidzein and equol concentrations

The concentrations of daidzein and equol in blood and urine were analysed by the time-resolved fluoroimmunoassay method; the detection limits of daidzein and equol in these assays were 2.0 nM and 3.3 nM, respectively [31,32].

### Statistical analysis

All values are expressed as mean ± SD, and P < 0.05 was considered significant. The differences in isoflavone and nutritional intake between equol producers and non-producers were examined by using unpaired Student’s t-test. The differences in the serum equol concentrations at baseline and after 2 weeks of intervention were examined by using paired Student’s t-test. Three-way ANOVA (factors of FOS intervention, time, and equol status) was performed to determine the effect of dietary intervention on the urinary equol to daidzein concentration ratios. The changes in the urinary equol to daidzein concentration ratios in each group were evaluated by repeated-measures ANOVA and the Tukey post hoc test. All analyses were performed by using SPSS (SPSS 11.0; SPSS Inc., Chicago, IL, USA).

## Results and discussion

At the beginning of the study, 43 subjects (25 equol producers, 18 non-producers) were recruited. Nine subjects withdrew because of personal reasons (5 subjects) and because they had taken oral antibiotics (4 subjects). The remaining 34 subjects completed the study, 21 (61.8%) were classified as equol producers, and 13 (38.2%) as non-producers. Characteristics of the subjects and their daily intake of isoflavones and nutrients at baseline are shown in Table 1. There were no major differences in age, height, weight, and body mass index between the equol producers and non-producers. The average daily intake of isoflavones from soy foods (other than isoflavone supplements) in each group ranged from 41.6–54.8 mg. Except for the fat intake, there were no significant differences between the daily intake of isoflavones and nutrients in the equol producers or non-producers. The average daily fat intake in equol producers (56.3 ± 13.7 g) was significantly higher compared to non-producers (49.1 ± 13.6 g). Epidemiological studies have been performed investigating the effects of habitual diet on equol production. Aldercreutz et al. found that the intake of total fat and meat and the dietary ratio of fat to fibre correlated with the urinary excretion of equol in a Japanese population [33], our result is consistent with this previous study. Whereas in a Western population, Rowland et al. reported that equol producers consumed significantly less energy as fat and significantly more energy as carbohydrate than equol non-producers [12]. The effects of habitual diet on equol production are controversial.

**Table 1:** **Equol producers and non**-**producers**: **subject characteristics and daily intake of isoflavones and nutrients**^**1**^

	**Equol producer**	**Equol non-producer**	***P*-value**^**2**^
	**n = 21**	**n = 13**	
Age (year)	53.1 ± 4.3	54.4 ± 3.8	0.168
Height (cm)	155.6 ± 4.7	155.2 ± 4.6	0.776
Weight (kg)	52.5 ± 7.4	51.5 ± 4.6	0.504
Body mass index (kg/m^2^)	21.6 ± 1.7	21.1 ± 2.3	0.328
Daily intake			
Isoflavones (mg)	41.6 ± 22.0	54.8 ± 48.7	0.195
Energy (kcal)	1820 ± 284	1796 ± 352	0.765
Protein (g)	72.0 ± 14.7	71.9 ± 14.5	0.986
Fat (g)	56.3 ± 13.7	49.1 ± 13.6	0.038
Carbohydrate (g)	246 ± 51	261 ± 54	0.264
Calcium (mg)	630 ± 167	639 ± 232	0.855
Dietary fibre (g)	17.3 ± 4.4	19.6 ± 8.4	0.212

^1^All values are mean ± SD.

^2^Differences in age, height, weight, body mass index, and daily isoflavone and nutrient intake between equol producers and non-producers were examined by using unpaired *t*-tests.

**Figure 2:**
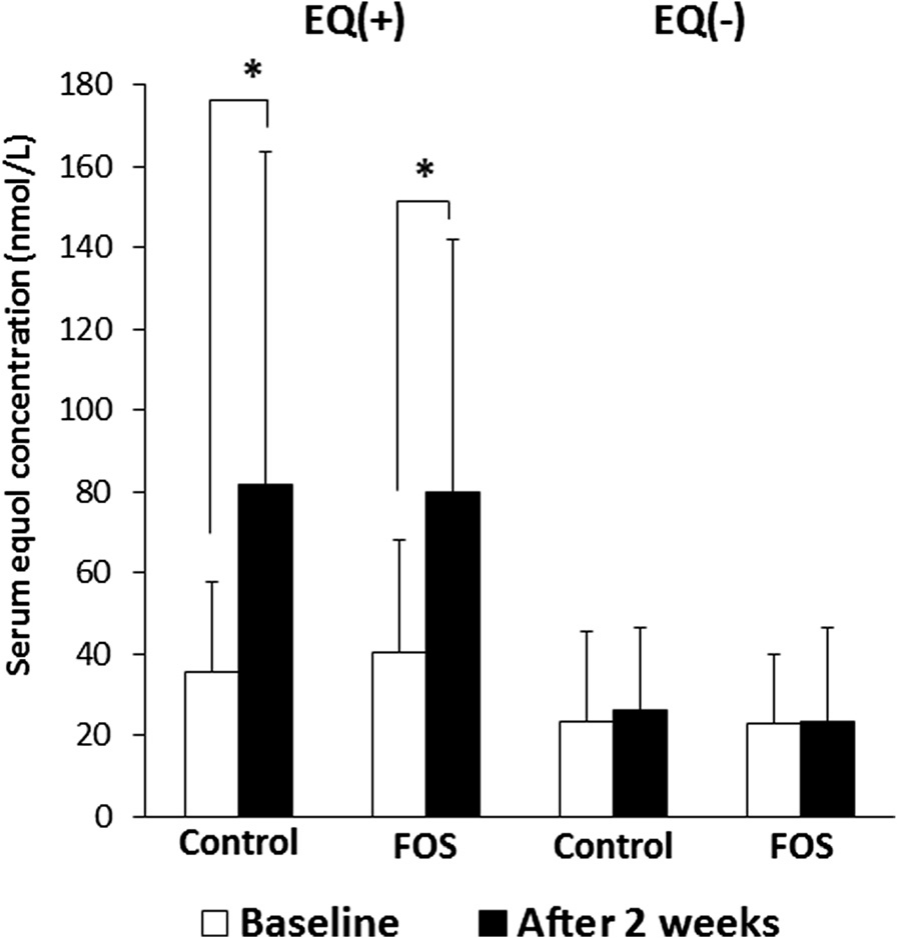
**Serum equol concentrations of different study groups at baseline and after 2 weeks of intervention.** EQ (+), equol producers; EQ (−), equol non-producers. Data is expressed as mean ± SD. Data was analyzed using a paired *t*-test. * Statistically significant (*P* < 0.05). The differences in the serum equol concentrations at baseline and after 2 weeks of intervention were examined using a paired Student’s *t*-test.

The serum equol concentrations in the equol producers and non-producers were 38.0 ± 24.8 nmol/L and 23.2 ± 19.0 nmol/L, respectively, and that of the equol producers was significantly higher than that of the non-producers (p = 0.008). Among the equol producers, serum equol concentrations in both the FOS and control groups significantly increased after 2-week of intervention (Figure 2). However, there were no significant differences in serum equol levels between the FOS and control groups in the equol producers. The similarly increased serum equol concentrations in the FOS and control groups among equol producers may have been stimulated by the isoflavone supplement. Among the equol non-producers, there were no significant differences in serum equol concentrations between the FOS and control groups at baseline and after 2 weeks of intervention. Thus, FOS intervention did not affect serum equol production as compared to sucrose intervention as control in the equol producers or non-producers who were treated with isoflavones in this study.

Setchell *et al*. have reported that expressing the product-precursor relationship as the urinary equol to daidzein concentrations ratio is a more reliable indicator of the conversion of daidzein to equol [34]. Among the equol producers and non-producers, 1- or 2-week FOS intervention had no effect on the capacity of colonic microbiota to produce equol (Table 2). Among the equol producers, urinary equol to daidzein concentration ratios in both the FOS and control groups was not significantly different after 1 week (p = 0.343 and p = 0.120, respectively) or 2 weeks (p = 0.578 and p = 0.432, respectively) compared with baseline. Similarly, in equol non-producers, FOS intervention did not affect the urinary equol to daidzein concentration ratios after 1 week (p = 0.794) or 2 weeks (p = 0.564) compared with baseline. In equol non-producers, no significant differences in urinary equol to daidzein concentration ratios in control group between baseline and after 1 week (p = 0.890) or 2 weeks (P = 0.383) were observed. Moreover, among the equol producers, urinary equol to daidzein concentration ratios in both the FOS and control groups was not significantly different between 1 and 2 weeks (p = 0.690 and p = 0.921, respectively). Similarly, among equol non-producers, urinary equol to daidzein concentration ratios in both the FOS and control groups was not significantly different between 1 and 2 weeks (p = 0.166 and p = 0.091, respectively).

**Table 2:** **Changes in urinary equol to daidzein concentration ratios in postmenopausal women**^**1**^

	**Equol producer**		**Equol non-producer**		***P*-value**						
	**(n = 21)**		**(n = 13)**		**3-way ANOVA**^**3**^						
					**Main effects**			**Interactions**			
	**FOS**^**2**^	**Control**	**FOS**	**Control**	**FOS**	**Time**	**Equol state**	**FOS × time**	**FOS × equol state**	**Time × equol state**	**FOS × time × equol state**
Baseline	−1.03 ± 0.46	−1.09 ± 0.733	−1.94 ± 0.30	−1.94 ± 0.44							
After 1 week	−0.77 ± 0.69	−0.74 ± 0.73	−2.04 ± 0.19	−2.07 ± 0.33	0.802	0.185	0.000	0.702	0.964	0.006	0.960
After 2 weeks	−0.92 ± 0.77	−0.82 ± 0.86	−1.83 ± 0.48	−1.62 ± 0.83	0.759	0.097	0.000	0.400	0.864	0.516	0.801
*P*-value^4^ (between baseline and after 1 week)											
	0.343	0.120	0.794	0.890							
*P*-value^4^ (between baseline and after 2 weeks)											
	0.578	0.432	0.564	0.383							
*P*-value^4^ (between 1 and 2 weeks)											
	0.690	0.921	0.166	0.091							

^1^Urinary equol to daidzein concentration ratios; (equol/daidzein) log. All values are expressed as mean ± SD.

^2^FOS, fructooligosaccharides.

^3^Three-way ANOVA (factors of FOS intervention, time, and equol status) was performed to determine the effect of dietary intervention on the urinary equol to daidzein concentration ratios.

^4^The changes in the urinary equol to daidzein concentration ratios in each group were evaluated by repeated-measures ANOVA and the Tukey post hoc test.

However, significant differences were observed in the interaction effect of time × equol state after 1 week of intervention (p = 0.006); however, there were no effects after 2 weeks of intervention (p = 0.516). One important reason for this observation could be the intervention time. Lampe et al. reported that isoflavone excretion did not differ according to the duration of soy intervention, whether 4 days or 1 month [26]. On the other hand, other studies have suggested that long-term exposure to isoflavone may change the usual plasma concentrations and urinary excretion of these compounds as result of altered metabolism [35,36]. After an initial increase, plasma concentrations of daidzein and genistein decreased when individuals consumed soy daily over a 2-week period [35]. Lu et al. reported that urinary recovery of genistein and daidzein decreased progressively over 4 weeks of daily soy ingestion but increased for equol [36]. Thus, such findings have not always been consistent among studies, and the intervention period that would elicit an isoflavone response is controversial.

In present study, there were no effects of 2-week FOS intervention on the capacity of colonic microbiota to produce equol. Supplementation with FOS and isoflavones for more than 2 weeks may be required in order to increase the capacity of colonic microbiota to produce equol. Another reason for this observation could be the diet. It is possible that a dietary intake of isoflavone may affect equol production [16,37]. Japanese diet typically contains higher amounts of soy products than the western diets, but the consumption of soy foods during the study was not controlled. The average daily total isoflavones (aglycone and conjugates) in this study ranged from 41.6 – 54.8 mg and isoflavone supplement contains 21 mg aglycone form. If dietary isoflavones may be sufficient isoflavone to saturate equol producer capacity, the supplement cannot elicit a response. Hence, the increased equol production in the equol producers after 1 week of intervention may correlate with isoflavones intake during this period. If a diet had low isoflavone content in our study, FOS supplementation may elicit a response by increasing the capacity of colonic microbiota to produce equol. FOS intervention and time and the interaction effects of FOS × time, FOS × equol state, and FOS × time ×equol state did not influence the urinary equol to daidzein concentration ratios significantly. Hence, our results suggest that a 2-week intervention with an FOS dosage of 5 g/day does not significantly modulate equol production both in equol producers and non-producers among postmenopausal Japanese women. Although we did not assess the gastrointestinal microbial activity in the present study, a daily FOS intake of 2.5–10 g for 2–4 weeks has been reported to have prebiotic effects [25,38]. Additionally, FOS have been reported to increase the number of faecal bifidobacteria [25,38], affecting the intestinal microbiotal balance [24]. Ohta et al. reported that FOS increased equol production from daidzein in OVX rats [39]. Thus, it is likely that FOS intake produced similar effects in the present study, although the effects on equol production were not significant. One possible reason for this observation could be that FOS intake of 5 g/day may not be sufficient to change and increase the number of intestinal microbiota required for a significant increase in equol production in humans. It was reported that the gastrointestinal tolerances of healthy male and female volunteers to FOS were 0.30 g/kg and 0.40 g/kg, respectively, and a FOS daily intake of up to 30 g was safe [40,41]. In this study, 5 g FOS was ingested per day. Therefore, if a much higher level of FOS were used, there may be effects on the intestinal microbiota of equol production. On the other hand, previous studies have reported that probiotics or prebiotic supplementation with soy does not affect equol production in American and Australian populations [26,27,42]. Although certain *Lactobacillus* and *Bifidobacteria* species have been suggested to play a role in the metabolism of daidzein to equol [23,43], the relevance of dietary-induced changes in gastrointestinal microbiota activity to isoflavone bioavailability is poorly understood. This suggests that further research addressing the roles of intestinal microbiota is required to determine the effects of FOS on equol production.

This is a pilot study, so there are several limitations in our study. Firstly, with regard to our Japanese subjects, dietary isoflavones during the study was not controlled. The Japanese diet contains higher amounts of soy products compared to Western diets. It is possible that a high dietary intake of isoflavone may have an effect on the capacity of equol production. Secondly, this study was carried out with small populations in each group. Sample size calculation, performed before the start of the study, showed that with 24 subjects in each group, a difference of 30 nmol/L between the means of urinary equol concentration could be shown with a power of 0.80 and two-side type 1 error of 0.05. Thus, it was seemed that 24 subjects in each group were enough for the study. However, this study included only 21 equol producers and 13 equol non-producers. One possible explanation for the non-significant effects of FOS treatment was the insufficient power as a result of the small sample size. Large scale studies with a similar design on statistically significant populations are essential to confirm our findings. Thirdly, it is a short-term analysis, and the subjects under review were adults whose gastrointestinal microbiotal composition is relatively stable. Some studies have suggested that long-term exposure to isoflavones may change the usual plasma concentrations and urinary excretion of these compounds as result of altered metabolism [36]. Hence, it is possible that the 2-week duration of this study was insufficient to alter the intestinal microbiota for which a longer trial period may be required. Additionally, the washout period was only 2 weeks, thus carryover effect might exist in our study. Based on the results derived, we intend to carry out a large-scale study in which the treatment period and FOS dose are modified, and dietary isoflavones are controlled.

## Conclusion

In conclusion, we have shown that a 2-week intervention with a daily dose of 5 g FOS does not significantly modulate the capacity of intestinal microbiota to produce equol in postmenopausal Japanese women, in either the equol producers or the non-producers treated with isoflavones in this pilot study. However, it may be possible to stimulate equol production with dietary conditions, especially probiotics such as *Lactobacillus* and *Bifidobacteria* species are added to a diet may be useful, on long-term intervention. Further large investigations that explore the roles of specific intestinal microbiota in equol production will enable the establishment of dietary conditions that are required to enhance equol production.

## Abbreviations

FOS: Fructooligosaccharides; ANOVA: Analysis of variance.

## Competing interests

The authors declare that they have no competing interests.

## Authors’ contributions

YI and MU conceived and designed the study. Substantial contributions to acquisition, analysis, and interpretation of data were made by YT, YI, FA, YK and MU. YT and YI drafted the manuscript. All authors read and approved the final manuscript.

## References

[B1] Schmitt E Dekant W Stopper H Assaying the estrogenicity of phytoestrogens in cells of different estrogen sensitive tissues Toxicol In Vitro 2001 15 433 439 10.1016/S0887-2333(01)00048-0 11566575

[B2] Matsumura A Ghosh A Pope GS Darbre PD Comparative study of oestrogenic properties of eight phytoestrogens in MCF7 human breast cancer cells J Steroid Biochem Mol Biol 2005 94 431 443 10.1016/j.jsbmb.2004.12.041 15876408

[B3] Barkhem T Carlsson B Nilsson Y Enmark E Gustafsson J Nilsson S Differential response of estrogen receptor alpha and estrogen receptor beta to partial estrogen agonists/antagonists Mol Pharmacol 1998 54 105 112 9658195 10.1124/mol.54.1.105

[B4] McCarty MF Isoflavones made simple - genistein's agonist activity for the beta-type estrogen receptor mediates their health benefits Med Hypotheses 2006 66 1093 1114 10.1016/j.mehy.2004.11.046 16513288

[B5] Hwang CS Kwak HS Lim HJ Lee SH Kang YS Choe TB Hur HG Han KO Isoflavone metabolites and their in vitro dual functions: they can act as an estrogenic agonist or antagonist depending on the estrogen concentration J Steroid Biochem Mol Biol 2006 101 246 253 10.1016/j.jsbmb.2006.06.020 16965913

[B6] Villa P Costantini B Suriano R Perri C Macri F Ricciardi L Panunzi S Lanzone A The differential effect of the phytoestrogen genistein on cardiovascular risk factors in postmenopausal women: relationship with the metabolic status J Clin Endocrinol Metab 2009 94 552 558 10.1210/jc.2008-0735 19017760

[B7] Taku K Melby MK Takebayashi J Mizuno S Ishimi Y Omori T Watanabe S Effect of soy isoflavone extract supplements on bone mineral density in menopausal women: meta-analysis of randomized controlled trials Asia Pac J Clin Nutr 2010 19 33 42 20199985

[B8] Khan SA Chatterton RT Michel N Bryk M Lee O Ivancic D Heinz R Zalles CM Helenowski IB Jovanovic BD Franke AA Bosland MC Wang J Hansen NM Bethke KP Dew A Coomes M Bergan RC Soy isoflavone supplementation for breast cancer risk reduction: a randomized phase II trial Cancer Prev Res 2012 52 309 319 10.1158/1940-6207.CAPR-11-0251 PMC3333836 22307566

[B9] Setchell KD Brown NM Lydeking-Olsen E The clinical importance of the metabolite equol-a clue to the effectiveness of soy and its isoflavones J Nutr 2002 132 3577 3584 12468591 10.1093/jn/132.12.3577

[B10] Lampe JW Karr SC Hutchins AM Slavin JL Urinary equol excretion with a soy challenge: influence of habitual diet Proc Soc Exp Biol Med 1998 217 335 339 10.3181/00379727-217-44241 9492344

[B11] Atkinson C Frankenfeld CL Lampe JW Gut bacterial metabolism of the soy isoflavone daidzein: exploring the relevance to human health Exp Biol Med 2005 230 155 170 10.1177/153537020523000302 15734719

[B12] Rowland IR Wiseman H Sanders TA Adlercreutz H Bowey EA Interindividual variation in metabolism of soy isoflavones and lignans: influence of habitual diet on equol production by the gut microflora Nutr Cancer 2000 36 27 32 10.1207/S15327914NC3601_5 10798213

[B13] Fujimoto K Tanaka M Hirao Y Nagata Y Mori M Miyanaga N Akaza H Kim WJ Age-stratified serum levels of isoflavones and proportion of equol producers in Japanese and Korean healthy men Prostate Cancer Prostatic Dis 2008 11 252 257 10.1038/sj.pcan.4501030 18180805

[B14] Akaza H Miyanaga N Takashima N Naito S Hirao Y Tsukamoto T Fujioka T Mori M Kim WJ Song JM Pantuck AJ Comparisons of percent equol producers between prostate cancer patients and controls: case-controlled studies of isoflavones in Japanese, Korean and American residents Jpn J Clin Oncol 2004 34 86 89 10.1093/jjco/hyh015 15067102

[B15] Arai Y Uehara M Sato Y Kimira M Eboshida A Adlercreutz H Watanabe S Comparison of isoflavones among dietary intake, plasma concentration and urinary excretion for accurate estimation of phytoestrogen intake J Epidemiol 2000 10 127 135 10.2188/jea.10.127 10778038

[B16] Nagata C Ueno T Uchiyama S Nagao Y Yamamoto S Shibuya C Kashiki Y Shimizu H Dietary and lifestyle correlates of urinary excretion status of equol in Japanese women Nutr Cancer 2008 60 49 54 18444135 10.1080/01635580701525885

[B17] Hedlund TE Maroni PD Ferucci PG Dayton R Barnes S Jones K Moore R Ogden LG Wähälä K Sackett HM Gray KJ Long-term dietary habits affect soy isoflavone metabolism and accumulation in prostatic fluid in caucasian men J Nutr 2005 135 1400 1406 15930444 10.1093/jn/135.6.1400

[B18] Katanoda K Matsumura Y National Nutrition Survey in Japan-its methodological transition and current findings J Nutr Sci Vitaminol 2002 48 423 432 10.3177/jnsv.48.423 12656220

[B19] de Kleijn MJ van der Schouw YT Wilson PW Adlercreutz H Mazur W Grobbee DE Jacques PF Intake of dietary phytoestrogens is low in postmenopausal women in the United States: the Framingham study(1–4) J Nut 2001 131 1826 1832 10.1093/jn/131.6.1826 11385074

[B20] Horn-Ross PL Lee M John EM Koo J Sources of phytoestrogen exposure among non-Asian women in California, USA Cancer Causes Control 2000 11 299 302 10.1023/A:1008968003575 10843441

[B21] Mulligan AA Welch AA McTaggart AA Bhaniani A Bingham SA Intakes and sources of soya foods and isoflavones in a UK population cohort study (EPIC-Norfolk) Eur J Clin Nutr 2007 61 248 254 10.1038/sj.ejcn.1602509 16943849

[B22] Tamura M Hori S Nakagawa H Lactobacillus rhamnosus JCM 2771: impact on metabolism of isoflavonoids in the fecal flora from a male equol producer Curr Microbiol 2011 62 1632 1637 10.1007/s00284-011-9904-6 21365446

[B23] Raimondi S Roncaglia L De Lucia M Amaretti A Leonardi A Pagnoni UM Rossi M Bioconversion of soy isoflavones daidzin and daidzein by Bifidobacterium strains Appl Microbiol Biotechnol 2009 81 943 950 10.1007/s00253-008-1719-4 18820905

[B24] Blaut M Relationship of prebiotics and food to intestinal microflora Eur J Nutr 2002 41 Suppl 1 I11 I16 12420111 10.1007/s00394-002-1102-7

[B25] Bouhnik Y Raskine L Simoneau G Paineau D Bornet F The capacity of short-chain fructo-oligosaccharides to stimulate faecal bifidobacteria: a dose–response relationship study in healthy humans Nutr J 2006 5 8 10.1186/1475-2891-5-8 16569219 PMC1448190

[B26] Lampe JW Skor HE Li S Wähälä K Howald WN Chen C Wheat bran and soy protein feeding do not alter urinary excretion of the isoflavan equol in premenopausal women J Nutr 2001 131 740 744 11238753 10.1093/jn/131.3.740

[B27] Larkin TA Astheimer LB Price WE Dietary combination of soy with a probiotic or prebiotic food significantly reduces total and LDL cholesterol in mildly hypercholesterolaemic subjects Eur J Clin Nutr 2009 63 238 245 10.1038/sj.ejcn.1602910 17940545

[B28] Larkin TA Price WE Astheimer LB Increased probiotic yogurt or resistant starch intake does not affect isoflavone bioavailability in subjects consuming a high soy diet Nutrition 2007 23 709 718 10.1016/j.nut.2007.06.010 17656069

[B29] Levy SB Levy SB, Novick RP Ecology of antibiotic resistance determinants Antibiotic resistance genes: Ecology, transfer and expression 1986 New York: Cold Spring Harbor Press 17 29

[B30] Richmond MH Drews J, Hogenauer G The survival of R plasmids in the absence of antibiotic selective pressure Topics in infectious diseases 1977 Berlin: Springer-Verlag 61 70

[B31] Wang GJ Lapcík O Hampl R Uehara M Al-Maharik N Stumpf K Mikola H Wähälä K Adlercreutz H Time-resolved fluoroimmunoassay of plasma daidzein and genistein Steroids 2000 65 339 348 10.1016/S0039-128X(00)00089-1 10802284

[B32] Brouwers E L'Homme R Al-Maharik N Lapcik O Hampl R Wahala K Mikola H Adlercreutz H Time-resolved fluoroimmunoassay for equol in plasma and urine J Steroid Biochem Mol Biol 2003 84 577 588 10.1016/S0960-0760(03)00071-2 12767283

[B33] Adlercreutz H Honjo H Higashi A Fotsis T Hämäläinen E Hasegawa T Okada H Urinary excretion of lignans and isoflavonoid phytoestrogens in Japanese men and women consuming a traditional Japanese diet Am J Clin Nutr 1991 54 1093 1100 1659780 10.1093/ajcn/54.6.1093

[B34] Setchell KD Cole SJ Method of defining equol-producer status and its frequency among vegetarians J Nutr 2006 136 2188 2193 16857839 10.1093/jn/136.8.2188

[B35] Barnes S Sfakianos J Coward L Kirk M Soy isoflavonoids and cancer prevention. Underlying biochemical and pharmacological issues Adv Exp Med Biol 1996 401 87 100 10.1007/978-1-4613-0399-2_7 8886128

[B36] Lu LJ Lin SN Grady JJ Nagamani M Anderson KE Altered kinetics and extent of urinary daidzein and genistein excretion in women during chronic soya exposure Nutr Cancer 1996 26 289 302 10.1080/01635589609514485 8910911

[B37] Song KB Atkinson C Frankenfeld CL Jokela T Wahala K Thomas WK Lampe JW Prevalence of daidzein-metabolizing phenotypes differs between Caucasian and Korean American women and girls J Nutr 2006 136 1347 1351 16614428 10.1093/jn/136.5.1347

[B38] Bouhnik Y Achour L Paineau D Riottot M Attar A Bornet F Four-week short chain fructo-oligosaccharides ingestion leads to increasing fecal bifidobacteria and cholesterol excretion in healthy elderly volunteers Nutr J 2007 6 42 10.1186/1475-2891-6-42 18053236 PMC2228298

[B39] Ohta A Uehara M Sakai K Takasaki M Adlercreutz H Morohashi T Ishimi Y A combination of dietary fructooligosaccharides and isoflavone conjugates increases femoral bone mineral density and equol production in ovariectomized mice J Nutr 2002 132 2048 2054 12097691 10.1093/jn/132.7.2048

[B40] Hata Y Nakajima K The relation of fructo-oligosaccharide intake and gastrointestinal symptom – The observations of no observed adverse effect level and 50% effective level Geriatr Med (in Japanese) 1985 23 817 828

[B41] Pharmacist's Letter/Prescriber's Letter editors Healthy food database (in Japanese) 2007 Tokyo: Daiichi publication 215

[B42] Nettleton JA Greany KA Thomas W Wangen KE Adlercreutz H Kurzer MS Plasma phytoestrogens are not altered by probiotic consumption in postmenopausal women with and without a history of breast cancer J Nutr 2004 134 1998 2003 15284389 10.1093/jn/134.8.1998

[B43] Yuan JP Wang JH Liu X Metabolism of dietary soy isoflavones to equol by human intestinal microflora–implications for health Mol Nutr Food Res 2007 51 765 781 10.1002/mnfr.200600262 17579894

